# Disparities in management of symptomatic osteoporotic vertebral compression fractures: a nationwide multidisciplinary survey

**DOI:** 10.1007/s11657-024-01454-8

**Published:** 2024-10-23

**Authors:** A. Weber, T. F. G. Vercoulen, E. Jacobs, A. T. Buizer, S. P. G. Bours, J. P. van den Bergh, R. M. Jeuken, S. M. J. van Kuijk, S. M. A. A. Evers, P. C. Willems

**Affiliations:** 1 Department of Orthopedics and Research School CAPHRI, Maastricht University Medical Center+, P. Debyelaan 25, 6229 HX Maastricht, The Netherlands; 2 Department of Orthopedics, University Medical Center Utrecht, Utrecht, The Netherlands; 3 Department of Orthopedics and Traumatology, University Hospital Ghent, Ghent, Belgium; 4 Department of Internal Medicine, Subdivision Rheumatology, CAPHRI Care and Public Health Research Institute, Maastricht University Medical Center +, P.O. Box 5800, 6202 AZ Maastricht, The Netherlands; 5 Department of Internal Medicine Research School NUTRIM, Maastricht University Medical Center+, Maastricht, The Netherlands; 6 Department of Internal Medicine, VieCuri Medical Center, Venlo, The Netherlands; 7 Department of Clinical Epidemiology and Medical Technology Assessment, Maastricht University Medical Center+, Maastricht, Netherlands; 8 Trimbos Institute-Netherlands Institute of Mental Health and Addiction, Utrecht, The Netherlands; 9 Department of Health Services Research, Care and Public Health Research Institute (CAPHRI), Faculty of Health, Medicine and Life Sciences (FHML), Maastricht University, Maastricht, The Netherlands

**Keywords:** Osteoporotic vertebral compression fracture, Osteoporosis treatment, Osteoporosis, Survey, Dissatisfaction, OVCF management

## Abstract

***Summary*:**

This nationwide multidisciplinary survey found dissatisfaction among physicians with current osteoporotic vertebral compression fracture care, revealing significant disparities in diagnosis, treatment, and follow-up practices. Issues include poor communication and differing guidelines. Improving interdisciplinary collaboration and standardized care strategies is essential for better patient outcomes.

**Purpose:**

This survey aims to assess current preferred care practices for symptomatic osteoporotic vertebral compression fractures (OVCF) in the Netherlands, focusing on guideline adherence, identifying knowledge gaps, and clarifying consensus and collaboration across medical disciplines in OVCF treatment.

**Methods:**

This cross-sectional study was conducted via Qualtrics (Provo, UT) using a self-administered online survey distributed to 238 general practitioners and physicians in orthopedics, traumatology, internal medicine, rheumatology, and geriatrics working at 51 hospitals in the Netherlands. The survey, conducted in Dutch, included 36 multiple-choice and two open questions and was accessible via an anonymous email link or QR code. General practitioners received additional questions specific to their role. Data was anonymized, stored securely, and analyzed using descriptive statistics in Microsoft Excel and SPSS (Version 24). Open-ended responses were coded and categorized. The survey was conducted prior to the publication of the updated Federation of Medical Specialists guidelines in 2024.

**Results:**

Physicians across various disciplines uniformly expressed dissatisfaction with current OVCF care. The survey highlighted significant disparities in diagnosis, treatment, and follow-up practices. A lack of communication between primary and secondary care providers and differing guidelines further complicate OVCF management. These issues point to considerable variation in clinical practice and gaps in interdisciplinary collaboration.

**Conclusion:**

Addressing the identified issues requires fostering interdisciplinary collaboration and creating cohesive care strategies. Ensuring access to diagnostic resources in both primary and secondary care and establishing coordinated care models promises more structured and standardized treatment. These steps are crucial for enhancing patient outcomes in OVCF management.

**Supplementary Information:**

The online version contains supplementary material available at 10.1007/s11657-024-01454-8.

## Introduction

Osteoporosis is a significant and widespread public health issue, primarily prevalent in postmenopausal women. Characterized by a disruption of the trabecular and cortical bone, the primary clinical consequence of osteoporosis is fragility fractures. In 2019, approximately 22.1% of women and 6.6% of men in the European Union were affected by osteoporosis [1–3]. Globally, this equates to hundreds of millions at risk of developing fragility fractures, a figure expected to rise as the population ages [2, 4].

Osteoporotic vertebral compression fractures (OVCFs) are among the most common fragility fractures, associated with high morbidity and mortality rates [5, 6]. Profoundly impacting patients’ quality of life, overall health, and independence, OVCFs often result in significant personal suffering, long-term functional decline, and substantial societal and economical costs [2, 7]. Following a symptomatic OVCF, patients face a 20% risk of subsequent fractures within a year [8], and in Europe alone, osteoporosis-related hip and spine fractures cause approximately 250,000 deaths annually [2]. Treatment strategies vary widely depending on country, clinic, medical specialty, and even individual physician [9, 10]. Recognizing the significance of OVCFs and in line with available international guidelines, such as the European guidance for the diagnosis and management of osteoporosis, the Dutch General Practitioner Society (Nederlands Huisartsen Genootschap, NHG) and the Federation of Medical Specialists (FMS) have provided a comprehensive, evidence-based framework for OVCF diagnosis and management, encompassing both conservative and surgical approaches [11–14]. Within these guidelines, the focus lies on diagnosis, determination of prevalent fractures using Vertebral Fracture Assessment, and prevention of future fractures [14].

While these guidelines may theoretically equip health care practitioners with strategies for proper management, they lack specificity and uniformity [11, 13, 15], leaving a considerable part of the treatment plan to the discretion of the attending physician.

The objective of this survey study was to provide insight into the current preferred care of patients with a symptomatic OVCF in the Netherlands while determining the interpretation of and adherence to the guidelines and identifying possible knowledge gaps. Additionally, this study aimed to clarify the extent of consensus and collaboration across different medical disciplines in the context of OVCF treatment.

## Methods

### Study population

This cross-sectional study utilized a self-administered online survey designed by a multidisciplinary team of medical specialists to assess individual medical professionals’ current preferred treatment strategy for patients with an OVCF in the Netherlands. Eligible participants were physicians and residents in orthopedic and traumatology, internal medicine, rheumatology, and geriatrics, as well as general practitioners. The survey was distributed to 45 general and six academic hospitals in the southern and western regions of the Netherlands.

### Study procedure

Requests for participation were distributed to all physicians at participating sites who are involved in OVCF care through their medical secretariats. General practitioners were invited to participate while attending a dedicated osteoporosis treatment training event. Two reminders were sent to all potential respondents. The survey was accessible via either an anonymous email link or a QR code at the training event. Due to General Data Protection Regulation restraints and high rotation rates within medical departments, the exact response rate could not be calculated.

The survey, conducted in Dutch via Qualtrics (Provo, UT) [16], included 36 multiple-choice and two open questions. For the purpose of international publication, both questions and answers have been translated (Appendix A). Respondents answered thirteen general questions about diagnosis and treatment, which led to further in-depth questions based on their answers. Additionally, background information on demographics and professional characteristics was collected. General practitioners received three additional questions specific to their role in the care pathway. Both general and in-depth questions were interpreted to evaluate knowledge of and adherence to existing guidelines and determine gaps and needs in the care pathway. Data were anonymized and stored securely on the hospital server; access was restricted to the research team.

### Statistical methods

The output for this study was generated using Qualtrics [16] software and analyzed using descriptive statistics. Responses to open-ended questions were coded and categorized using the Statistical Package for the Social Sciences (SPSS; Version 24, IBM, NY, USA).

## Results

Fifty-one hospitals were approached, 253 physicians initially responded, and a total of 238 physicians completed the survey (94% completion rate). Thirty-nine percent of respondents were general practitioners, 36% were surgeons (trauma surgeons or orthopedic surgeons), 25% were physicians in the field of internal medicine (internists, rheumatologists, geriatricians), and 1% were emergency doctors. Of all respondents, 89% of general practitioners, 82% of surgeons, and 92% of internists indicated that care for OVCF patients was insufficient. Respondent demographics including experience and work environment are provided in Table 1.

**Table 1:** Descriptive statistics—categorical variables

	*Medical discipline*								
***Experience***	***General medicine***	***Traumatology***	***Orthopedic surgery***	***Internal medicine***	***Rheumatology***	***Clinical geriatrics***	***Emergency medicine***	***Unknown***	***Total***
*Residents*	7	1	12	3	0	0	0	1	**24**
*0–5 years*	42	7	11	9	4	3	1	5	**82**
*6–10 years*	13	2	19	5	3	0	2	4	**48**
*11–15 years*	6	2	8	10	2	3	0	2	**33**
*16–20 years*	6	1	3	4	1	2	0	2	**19**
*21–25 years*	4	4	3	2	0	1	0	4	**18**
*More than 25 years*	7	2	3	0	0	1	0	3	**16**
***Setting***		***Traumatology***	***Orthopedic surgery***	***Internal medicine***	***Rheumatology***	***Clinical geriatrics***	***Emergency medicine***		***Total***
*Academic*		8	21	9	4	2	0		**44**
*General hospital*		11	38	24	6	8	3		**90 (62)**
***Regional/local protocols***	***General medicine***	***Traumatology***	***Orthopedic surgery***	***Internal medicine***	***Rheumatology***	***Clinical geriatrics***	***Emergency medicine***	***Unknown***	***Total***
*Yes*	21	12	35	11	5	4	2	3	**93**
*No*	59	6	23	13	2	3	0	5	**111**
***Follow NHG/FMS guidelines***	***General medicine***	***Traumatology***	***Orthopedic surgery***	***Internal medicine***	***Rheumatology***	***Clinical geriatrics***	***Emergency medicine***		***Total***
*Yes*	74	14	43	16	7	7	1	4	**166**
*No*	1	2	9	1	0	0	1	2	**16**
*Other*	5	0	0	0	0	0	0	0	**5**

Data in bold emphasis indicate the total per category or question

### Diagnosis

**Fig. 1:**
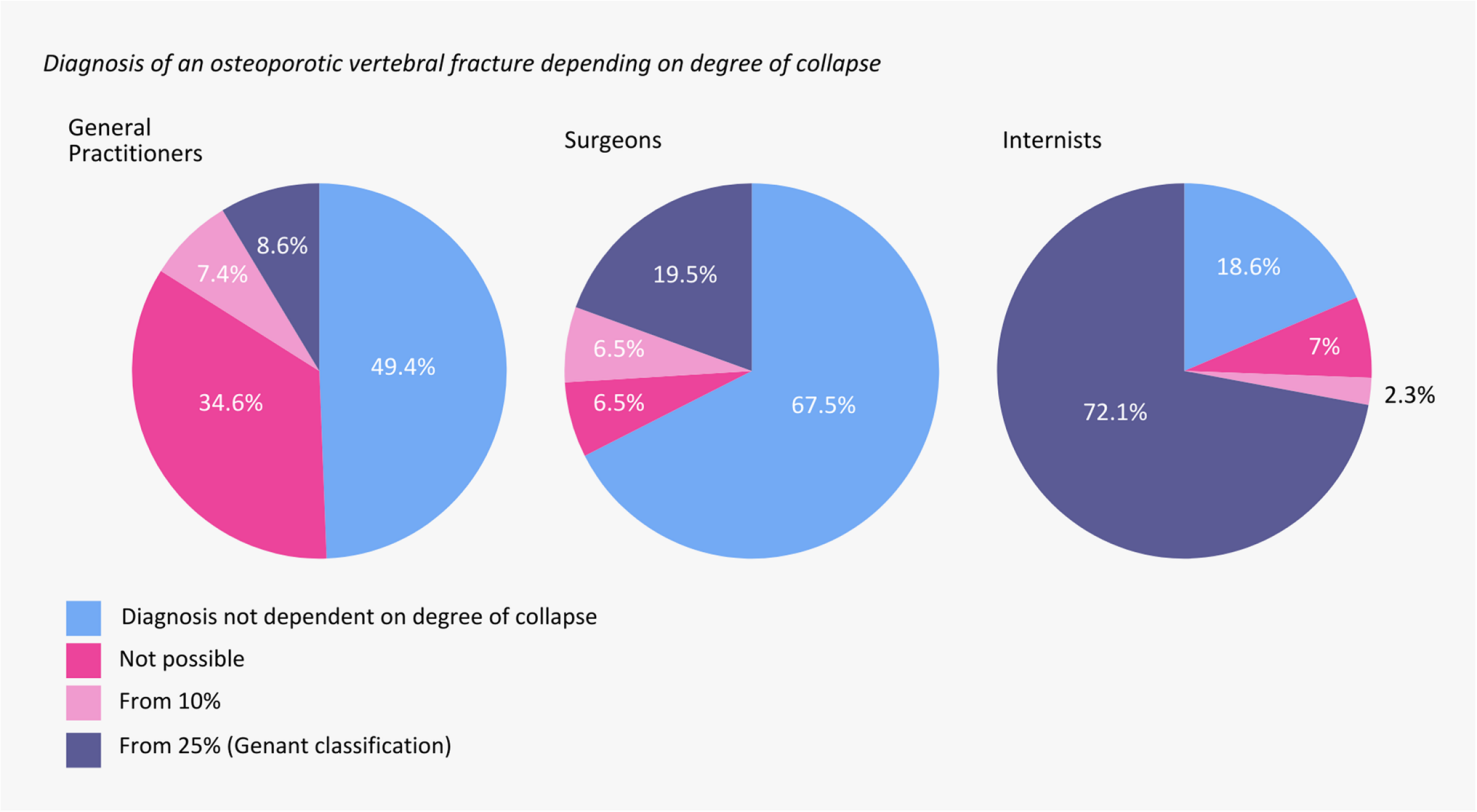
Pie chart displaying whether medical specialists make a diagnosis of an osteoporotic vertebral fracture depending on degree of collapse. The question respondents received was “From what degree of collapse do you diagnose an osteoporotic vertebral fracture?”

Respondents indicated that the most important factors in deciding to obtain conventional radiographs in patients suspected of having a symptomatic OVCF were patient history (95%), localization, origin, and intensity of the pain (94%), and the onset, duration, and course of symptoms (94%). The least important factors were sex and level of (in)activity of the patient. When asked about the diagnosis of a vertebral fracture, there was no consensus among medical specialties about the degree and relevance of vertebral body collapse (Fig. 1). General practitioners and surgeons mostly stated that the diagnosis is unrelated to the degree of collapse of the vertebral body, whereas internists mainly indicated that the diagnosis OVCF can only be made in case of at least 25% collapse.

In case of an acute onset of symptoms, such as following low-energy trauma, 40% of respondents working in a hospital setting indicated they always use additional imaging besides radiographs, in the form of a CT scan or an MRI, while 36% never obtain additional imaging (29% of surgical specialties and 47% of internal medicine). Almost half of all surgeons (47%) will request an additional CT scan and 2.5% an MRI; 15% of internists request a CT scan and 3.8% an MRI. The vast majority (82%) of general practitioners indicate that they never obtain additional imaging. Respondents with more professional experience were more likely to request an additional CT scan (30% of respondents with more than 15 years of experience versus 17% for doctors with less than 15 years of experience).

If osteoporosis is suspected, 76% of the respondents perform a dual-energy X-ray absorptiometry (DXA) scan. Surgeons (49%) request a DXA scan less often than general practitioners (89%) or respondents working within internal medicine (84%). When taking referral to other specialists into account, 83% of surgeons either requested a DXA themselves or indicated they referred to other specialists as part of standard treatment. Interestingly, more experienced physicians indicated that they are more inclined to request a DXA scan than those still at the start of their careers (72% vs. 54%).

Important factors in the decision to make a DXA scan are a recent fracture (98%), metabolic bone disease (96%), and advanced age (92%). The least important factors are the FRAX algorithm (42%), reduced mobility (50%), and rheumatoid arthritis (56%).

### Treatment and management

Forty-six percent of the respondents expressed that within their region a regional or local consensus exists for OVCF treatment. In case of a documented new OVCF, as reported by the radiologist, 69% of the general practitioners in the Netherlands will treat these patients themselves as opposed to 19% who refer to a medical specialist and 4.7% where radiologists will automatically refer to secondary care.

**Fig. 2:**
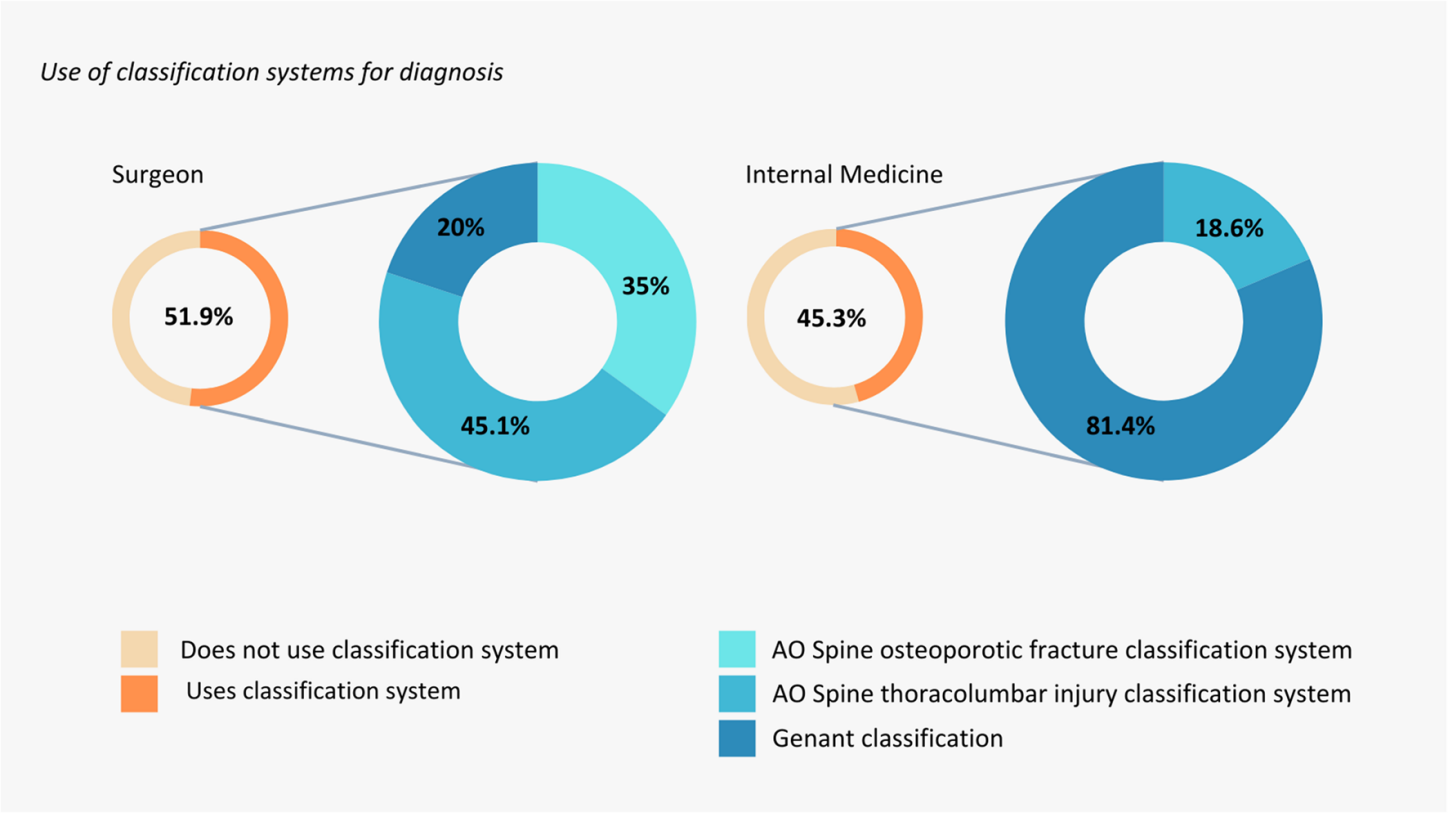
Actual use of classification systems by respondents and preferred classification systems. Respondents were asked “Do you use a classification system to characterize the osteoporotic fracture?” If response was “Yes,” respondents were asked “What classification system do you use?” Responses to use of classification systems can be seen on the left hand sides of the graphs and answers to which specific classification systems can be seen on the left. Responses were analyzed and are shown based on medical specialty

When asked whether they followed the current NHG or FMS guidelines for treatment of fracture prevention, 87% of general practitioners, 70% of surgeons, and 60% of internists indicated that they did [11, 13]. A total of 53% of the surgeons used a classification system for osteoporotic fractures and 45% of the internists. For the surgeon subgroup, 76% used the AO Spine Classification Systems and 21% the Genant classification [17]. Of the internist subgroup, this was 4% and 83% respectively (Fig. 2). Of the users of a classification system, around 70% indicated that the classification system influenced the treatment plan.

**Fig. 3:**
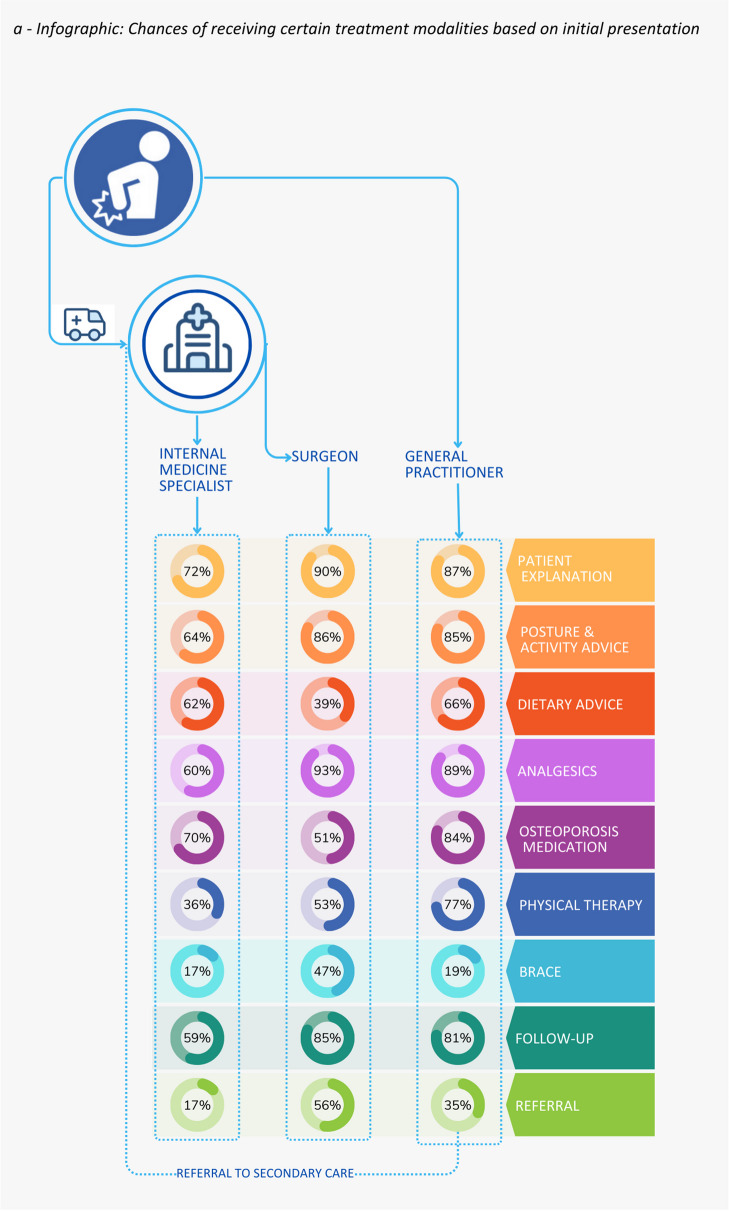

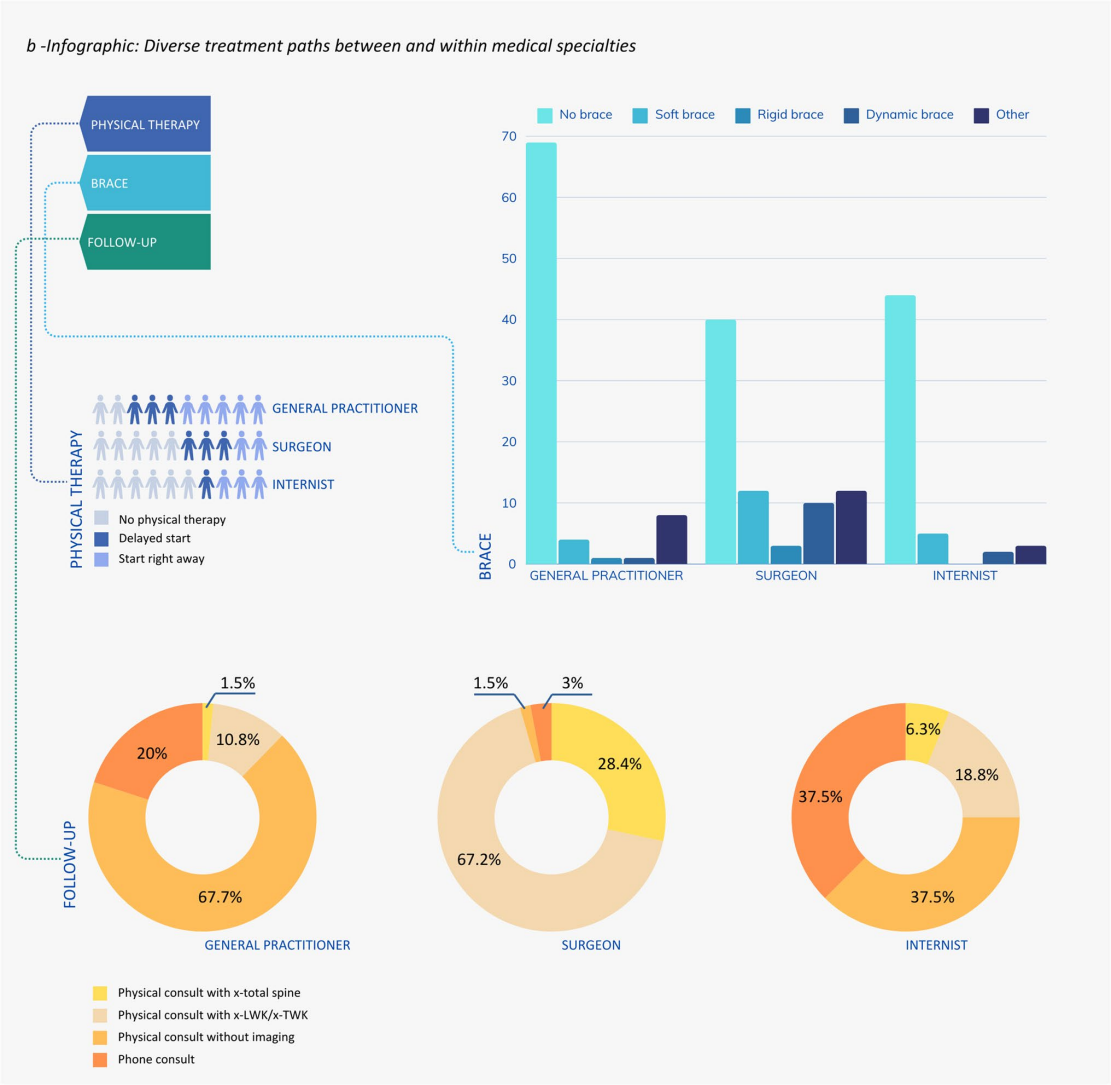
**a** Infographic on treatment likelihood based on patient presentation and primary caregiver. Patients with acute OVCF either visit the emergency department, where they are treated by an internal medicine specialist or surgeon, or they see their general practitioner. Respondents were asked about standard treatment components in their practice. **b** Infographic on treatment likelihood based on patient presentation and primary caregiver. Respondents indicating they prescribed physical therapy, a brace, or planned follow-up outpatient visits were asked several follow-ups concerning physical therapy initiation, type of brace, and type of outpatient consultation with or without additional imaging

Patient explanation (96%), painkillers (95%), and mobility-related advice (91%) are the most commonly reported treatment modalities incorporated in the standard care of patients presenting with less than 6 weeks of symptoms. Physical therapy (68%), dietary advice (64%), or a brace (34%) were less commonly utilized. Figure 3a illustrates the likelihood of receiving specific treatment dependent on where patients present themselves, taking into account the variability across medical specialties and the lack of consensus within these specialties regarding the treatment plan. Figure 3b presents several sub-analyses pertaining to the prescription of braces, physical therapy, and follow-up procedures. As can be seen in these figures, the choice for certain treatment modalities varies widely both between and within specialties. Referral to second-line care or another specialty was reported by 35% of general practitioners, 56% of surgeons, and 17% of internists. Osteoporosis medication was prescribed by 84% of general practitioners, 51% of surgeons, and 70% of internists. Patient monitoring was common for 81% of general practitioners and 85% of surgeons, as opposed to 42% of internists. Follow-up visit protocols also differ among specialties. Upon indicating they planned follow-up visits, general practitioners indicated they prefer to plan physical consultations without additional imaging (68%), surgeons prefer physical consultations with either radiology of the total spine (67.2%) or lumbosacral/thoracic vertebral column (28.4%), and internists prefer either a phone consultation (37.5%) or physical consultation without additional imaging (37.5%). Among those prescribing physical therapy, 60% of general practitioners and 74% of internists recommend immediate physical therapy, while 54% of surgeons suggest delaying. Braces are prescribed by 46.8% of surgeons and 17% of internists, but there is no clear preferred type.

### Bottlenecks and opportunities in optimizing care

**Fig. 4:**
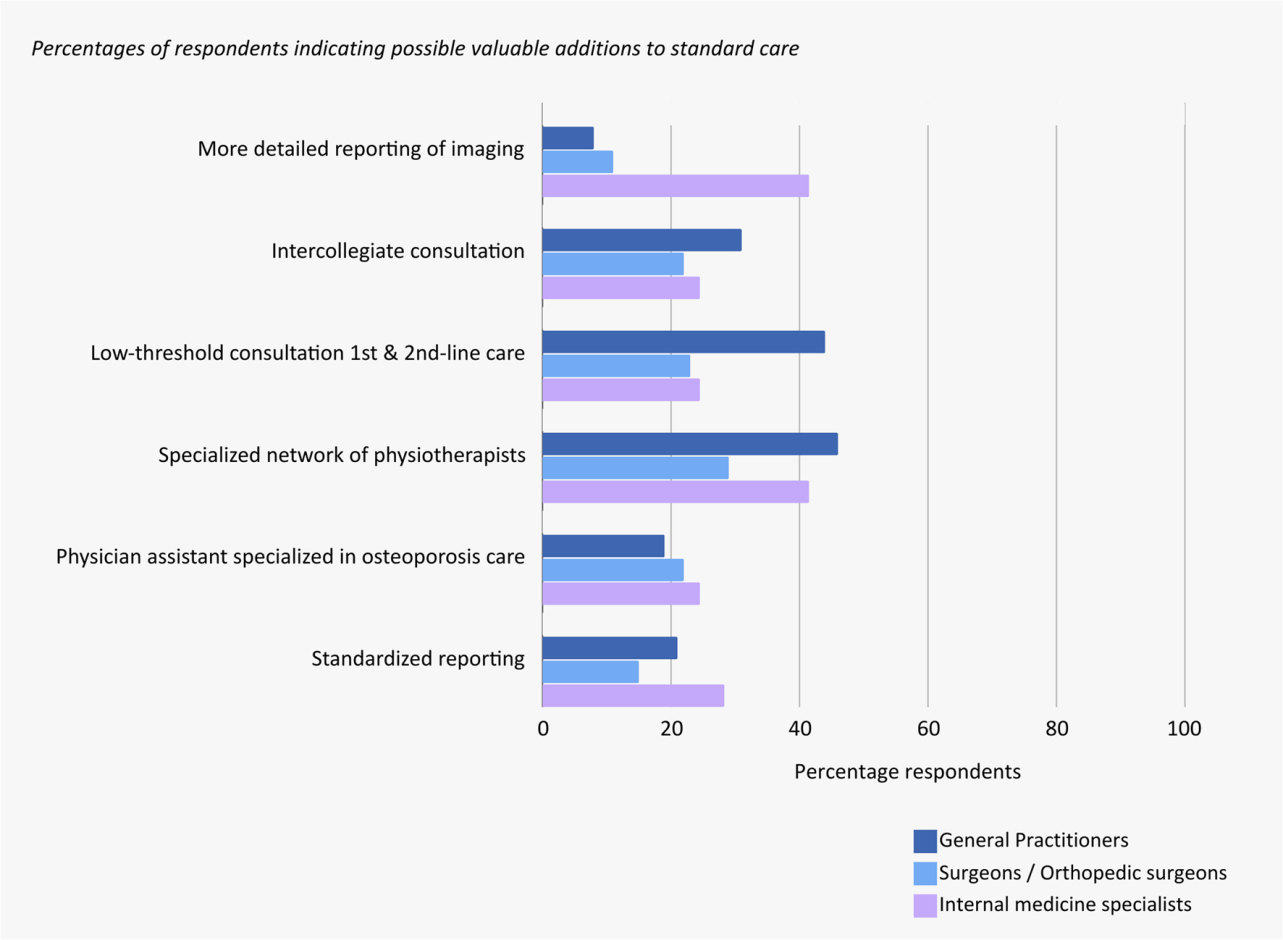
Bar graph showing the answers to questions regarding potential additions to care. Internal medicine specialists indicated they would be most interested (42%) in “More detailed reporting of radiographic images,” whereas general practitioners expressed more interest (44%) in “Low-threshold consultation 1st and 2nd line care.” Both general practitioners (46%) and internal medicine specialists (42%) would consider having a “Specialized network of physiotherapists” a valuable addition to care

Respondents identified a specialized physiotherapist network and increased consultation opportunities between general practitioners and specialists as the most valuable additions to current OVCF treatment options and key areas for optimizing care (Fig. 4).

**Fig. 5:**
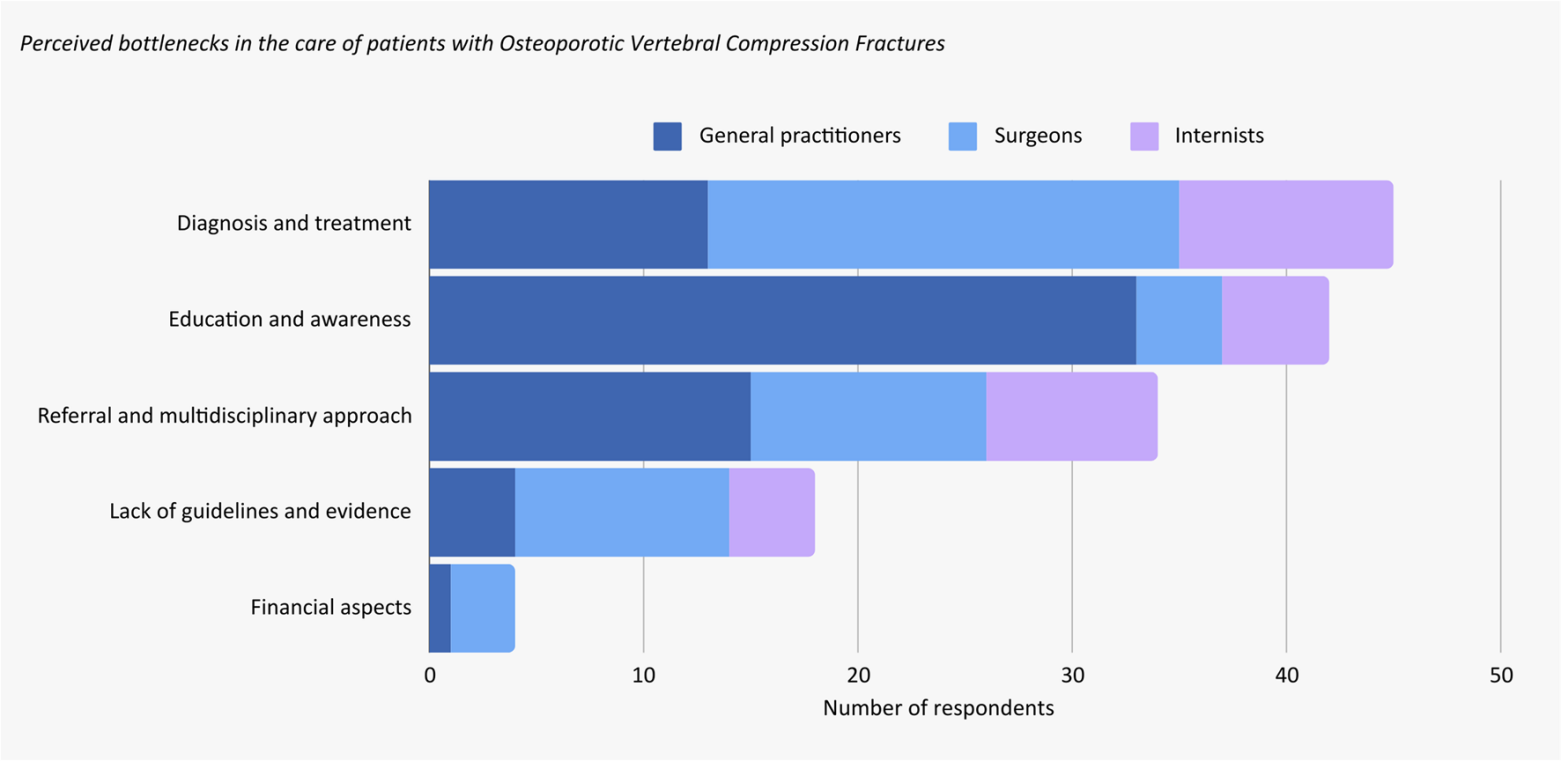
Bar graph showing the categorized answers on an open question regarding bottlenecks surrounding the care of patients with OVCF. Diverse responses were provided, which were categorized into five themes: Diagnosis and treatment, Referral and multidisciplinary approach, Financial aspects, Lack of guidelines and evidence, Education and awareness. The answers are presented per specialization of physicians. General practitioners mainly mentioned issues concerning education and treatment, whereas surgeons mainly mentioned bottlenecks in the field of diagnosis and treatment

When given an open question regarding the bottlenecks in the care of patients with OVCF, a variety of answers were given that could be categorized into five different categories (Fig. 5).

## Discussion

This survey among Dutch health care professionals unveils a considerable heterogeneity both within and between medical disciplines in the management of patients with osteoporotic vertebral compression fractures. In accordance with the ambiguity seen in diagnosis and treatment, there is an almost unanimous consensus among respondents that care for patients with OVCF is inadequate. If addressed correctly, improvement of bottlenecks as highlighted in this survey could potentially lead to a more uniform and effective care pathway.

Patients faced with an acute symptomatic OVCF typically pursue one of two clinical pathways: either seeking initial evaluation at an emergency department, leading to subsequent management in secondary care, or seeking consultation with a general practitioner. Regardless of who becomes the primary caregiver, it is imperative patients should receive the same quality of care wherever they initially present. Currently, physicians in primary and secondary care adhere to their own distinct clinical practice guidelines which contain conflicting directives regarding diagnosis and (pharmaco-)therapy [11, 13]. Our survey shows that the majority of general practitioners prefers to manage OVCF cases autonomously.

When diagnosing an OVCF, general practitioners rely on the report of the radiologist, the BMD, and a patient’s risk profile to determine whether to prescribe osteoporosis medication. These reports often vary in descriptive terms for the OVCF, leading to a lack of uniformity. As described by Genant et al., a grade 1 vertebral fracture is defined by a decrease in height of ≥ 20% [17]. According to the FMS guidelines, medication is indicated from grade 2 fractures, characterized by a height loss of ≥ 25% [11, 17, 18]. Although guidelines encourage all physicians to use the Genant score, this classification system offers limited value in assessing mechanical stability or symptomatology, making it less relevant for surgical specialists. When respondents were asked whether they presently use a classification system, approximately half of all surgeons and internists answered affirmatively. As could be expected, surgeons prefer more mechanically oriented classification systems such as the AO Spine classification systems [19, 20]. Moreover, the majority of surgeons indicated that the diagnosis of OVCF is not determined by the degree of vertebral collapse. The discrepancies concerning classifying OVCFs underline a noticeable incongruity between current guidelines and the realities of daily clinical practice. A potential solution to ensure adherence to guidelines could be the incorporation of standardized radiology reports, using the cutoff value of > 25% vertebral body height loss. By implementing this grading system and employing clear and unambiguous reporting practices, radiologists can play a pivotal role in facilitating optimal treatment [18, 21].

Another notable discrepancy in the diagnosis of OVCF involves the decision to use additional imaging. Approximately half of the surgeons surveyed routinely request additional imaging, predominantly computed tomography (CT). While reasons motivating the acquisition of CT scans or MRI scans were not explored in our study, the prevalence likely reflects regional variations with respect to availability and preference. Although warranted in specific cases of diagnostic uncertainty, high energy trauma, neurological symptoms, or pathological fractures, routine use of additional imaging may impose a significant burden on patients and health care systems in terms of inconvenience and costs [22–27]. Current clinical guidelines prioritize preventing secondary fractures by addressing underlying osteoporosis. Dutch guidelines align with international guidelines, emphasizing prevention with clear, albeit incongruent, pharmacotherapy flowcharts. Ambiguity persists, however, concerning the management of symptoms caused by the fracture itself. The American Academy of Orthopedic Surgeons (AAOS) offers recommendations concerning care and prevention, although evidence for most of these, including bed rest, opioids, analgesics, supervised or unsupervised exercise programs, or treatment with a brace, is considered weak or inconclusive [15]. A 2017 review concerning OVCF care found that advice for diagnosis and therapy was generally inconsistent and efforts are needed to improve the quality of the majority of guidelines [28]. From a broader perspective, this issue extends beyond the management of OVCF and encompasses the overall approach to osteoporosis and high fracture risk management. This underscores the need for a more harmonized and unified approach to guideline development. As stated by the AAOS and echoed by our respondents, the lack of robust evidence for treatments like bracing or physical therapy underscores the need for higher-level evidence to refine guidelines and establish gold standards for fracture management. Contradictory advice provided by different medical specialty societies contributes to persistent confusion, eventually resulting in suboptimal patient care. Concrete efforts must be made, both now and moving forward, to harmonize guidelines and ensure patient care is optimized in the long term.

Exemplary of this ambiguity is treatment using orthoses. While favorable outcomes in terms of pain, function, and well-being have been reported with a dynamic brace, insufficient evidence exists to strongly recommend bracing for symptomatic OVCF [15, 29]. Nevertheless, nearly half of the surgeons prescribe a brace upon presentation. Indeed, the debate surrounding the efficacy of bracing and potential application as therapeutic intervention following OVCF has been reignited by a multicenter randomized controlled trial currently being conducted in the Netherlands [30]. This survey also highlights a significant divergence regarding physical therapy referrals and timing among respondents. Several studies have indicated that exercise interventions may successfully preserve or increase bone mineral density (BMD) [31–34]. These programs have repeatedly been proven to be successful in preventing injury and reducing the number of falls and fractures in populations with increased risk of osteoporosis [31, 35, 36]*.* Less than 40% of individuals in the general public aged 65 and older engage in 150 min of moderate-intensity exercise and two additional sessions of bone-strengthening activities per week, as advised in the Dutch physical activity guidelines, suggesting potential benefits for OVCF patients through exercise interventions or physical therapy [37, 38]. Exercise programs for osteoporosis patients should, at a minimum, include balance and muscle-strengthening exercises [39]. Relying solely on the prescription of physical therapy, as favored by surgeons, or osteoporosis medication, as more commonly practiced by internists, fails to fully realize the potential for optimal patient outcomes [40].

In spite of the dissatisfaction with available guidelines and care options, the Netherlands is performing favorably compared to many other European countries [3]. When looking at the clinical and economic burden of osteoporotic fractures within the EU, the Netherlands presents as one of the lowest-burden, highest-provision countries and provides full reimbursement for treatment [3]. Taking the responses to this survey into account, it becomes clear however that much is still to be won. Since the respondents were invited to participate in the survey, updated guidelines for Dutch medical specialists have been published [11]. These guidelines, prompted in part by disparities as identified in this survey, provide treating physicians with enhanced guidance in terms of fracture management.

To monitor whether proper implementation and use of these guidelines will actually lead to the intended changes, it would be valuable to repeat this survey in a few years’ time. Standardizing both screening and referral protocols as well as prescription, treatment, and follow-up practices is an essential step towards improving patient outcomes. Further research is needed to evaluate the impact of physical therapy, medication, bracing, and the combination thereof, as well as the role of CT scans in standard care. This survey underscores the need for studies to determine whether increased education, streamlined care, enhanced cooperation, and more uniform policies are necessary. Registries and qualitative studies might help answer these questions.

### Limitations

Inherent to the nature of a survey study, the major limitation of the present study is its subjective outcomes. The results of the present study should be interpreted as the preferred management strategy of Dutch physicians involved in the care of OVCFs. Due to the General Data Protection Regulation and high rotation rate within medical departments, it was not possible to calculate the exact response rate. Despite this shortcoming, the contributions made by the 238 respondents from primary and secondary care can be considered to be a substantial and representative response since these respondents, as compared to non-respondents, are those health care providers directly involved in the management of patients with an OVCF. Furthermore, this survey only included physicians with a direct treatment relationship with the patient. Radiologists and nuclear medicine specialists were not included in this study. Consequently, this study could not provide sufficient information regarding the uniformity of reporting and evaluation by radiologists and nuclear medicine specialists and the factors involved.

## Conclusion

In this survey, physicians across different disciplines responsible for treating patients with an OVCF uniformly and overwhelmingly expressed dissatisfaction with current care, citing disparities in diagnosis, treatment, and follow-up practices. The lack of consultation between primary and secondary care, along with separate differing guidelines, further complicates OVCF management. While the optimal approach remains undetermined, fostering interdisciplinary collaboration and creating cohesive care strategies are crucial. Ensuring access to diagnostic resources in primary and secondary care and establishing coordinated and harmonized care models offers promise for more structured and standardized treatment, enhancing patient outcomes.

## Supplementary Information

Below is the link to the electronic supplementary material.

Supplementary file1 (DOCX 21 KB)

## Data Availability

Data is available from the corresponding author upon reasonable request.
