# Supplementary material for: Disparities in management of symptomatic osteoporotic vertebral compression fractures: a nationwide multidisciplinary survey
Source: Arch Osteoporos. 2024 Oct 23;19(1):101. doi: 10.1007/s11657-024-01454-8 (PMC11499376; doi:10.1007/s11657-024-01454-8)
Supplement: Supplementary file 1 — Supplementary file1 (DOCX 21 KB) [file 11657_2024_1454_MOESM1_ESM.docx]

Q1. Within which specialty do you work?

- General medicine
- Surgery
- Orthopedics
- Internal medicine
- Rheumatology
- Clinical geriatrics
- Emergency medicine
- Other

Q2. How long have you been working within your current discipline?

- Still in training
- 0-5 years
- 6-10 years
- 11-15 years
- 16-20 years
- 21-25 years
- More than 25 years

*Display this question: If within which specialty are you working ≠ general medicine*Q3. In which setting do you work?

- Academic
- Peripheral

*Display this question: If within which setting are you working = peripheral*Q4. If peripheral, do you work at a STZ (Collaborating Top Clinical Hospital)?

- Yes
- No

*Display this question: If within which specialty are you working = general medicine*Q5. When the result of the photograph shows a new osteoporotic vertebral fracture in a patient aged 50+, what is your policy?

- I remain in control of the treatment plan/follow up
- I refer the patient for treatment in secondary care
- The radiologist automatically forwards these patients to secondary care
- Other, namely _________

*Display this question: If when the result of the photograph shows a new osteoporotic vertebral fracture.. = I refer the patient for treatment in secondary care*Q6. I refer to the

- Orthopedic surgeon
- Pain specialist
- Internist
- Trauma surgeon
- Rheumatologist

Q7. On which factors do you base the choice to send patients with suspected vertebral fractures to radiology for x-ray diagnosis? (Yes/No)

- Gender
- Age
- Duration, onset and course of complaints
- Localization, nature and intensity of the pain
- Influence of (in)activity
- Patient concerns/wants
- Past history (including previous fractures)
- Degree of impairment in daily functioning
- Other ______________

Q8. In addition to conventional radiographs, do you use additional imaging for osteoporotic vertebral fractures in the acute setting (e.g. after low-energy trauma)?

- No
- Yes, a CT scan
- Yes, an MRI scan
- Other, namely ______________

*Display this question: If within which specialty are you working = general medicine*Q9. Do you have x-rays taken again for persistent back pain despite previous x-rays not indicating a vertebral fracture?

- Yes
- No
- Other, namely______

Q10. Do you request a DEXA scan for diagnosis of suspected osteoporosis?

- Yes
- No

*Display this question: If do you request a DEXA scan for diagnosis of suspected osteoporosis? = yES*Q11. Do you see the following factors as important indications for requesting a DEXA scan? (YES/No)

- Age
- Recent fracture including hip and/or vertebral fracture
- Reduced mobility
- Rheumatoid arthritis
- >1x fall last year
- Has condition associated with osteoporosis
- Familial history osteoporosis
- According to FRAX-algorithm
- Use of glucocortcosteroids>7.5mg/day
- Weight

Q12. Does your practice use an automated system that alerts/automatically refers when someone meets the referral criteria for a DEXA scan?

- Yes
- No

Q13. From what degree of collapse do you diagnose an osteoporotic vertebral fracture?

- From 10%
- From 15%
- From 25%
- Diagnosis is not determined by degree of collapse
- Not possible

Q14. Do you use a classification system to characterize the osteoporotic fracture?

- Yes
- No

*Display this question: If Do you use a classification system to characterize the osteoporotic fracture? = Yes*Q15. What classification system do you use?

- AO Spine thoracolumbar injury classification system
- AO Spine osteoporotic fracture classification system
- Genant classification
- Other, namely _____________

*Display this question: If do you use a classification system to characterize the osteoporotic fracture? = Yes*
Q16. Does the classification system influence your treatment plan?

- Yes
- No
- Other, namely______

Q17. Have regional/local working agreements been established regarding treatment of vertebral fractures?

- Yes, created by_____________
- No

Q18. Do you follow the current national NHG/FMS guidelines for fracture prevention treatment after a vertebral fracture?

- Yes
- No
- Other, namely______

Q19. Are the following elements part of the standard treatment of a patient with an osteoporotic vertebral fracture and symptoms for less than 6 weeks? (Yes/No) *Multiple answers possible.*

- Explanation
- Advice regarding posture and movement
- Nutritional advice
- Pain relief
- Osteoporosis medication
- Physical therapy
- Brace
- Follow-up
- Referral to secondary care/other specialist
- I don’t see patients with a recent osteoporotic vertebral fracture

*Display this question: If are the following elements part of…= pain relief*
Q20. Please specify below which pain relief is part of the standard treatment in your practice for a patient with an osteoporotic vertebral fracture and symptoms for less than 6 weeks. *Multiple answers possible.*

- According to pain ladder WHO
- Only paracetamol
- NSAIDS if there are no contraindications
- Oral opiates
- Transcutaneous opiates
- Other, namely________

*Display this question: If are the following elements part of…= Osteoporosis medication*
Q21. Please specify below which osteoporosis medication is part of the standard treatment in your practice for a patient with an osteoporotic vertebral fracture and symptoms for less than 6 weeks. *Multiple answers possible.*

- Androgens
- Bisphosphonates
- Bisphosphonates, combination preparations
- Calcium with vitamin D, combination preparations
- Zoledronic acid intravenously
- Calcium regulators: Denosumab, Romosozumab, Teriparitide
- Calcium carbonate
- Estrogen with progesteron, postmenopausal
- Estrogen receptor modulaters
- Estrogens
- Vitamin D and analogs
- Other, namely_________

*Display this question: If are the following elements part of…= physical therapy*
Q22. Specify below when you would recommend starting physical therapy as part of the standard treatment in your practice for a patient with an osteoporotic vertebral fracture and symptoms for less than 6 weeks.

- Start immediately
- Start at a later date

*Display this question: If are the following elements part of…= brace*
Q23. Specify below which brace is part of the standard treatment for patients with an osteoporotic vertebral fracture and symptoms for less than 6 weeks.

- Lumbar brace
- Dekyphotic brace
- Three-point brace
- Dynamic brace (including Osteolind, Spinova Osteo, Spinomed)
- Other, namely______________

*Display this question: If are the following elements part of…= follow-up*
Q24. After how many weeks do you schedule a follow-up appointment as part of standard treatment for patients with an osteoporotic vertebral fracture and symptoms for less than 6 weeks?

*Display this question: If are the following elements part of…= follow-up*
Q25. What follow-up appointment do you schedule as part of standard treatment for patients with an osteoporotic vertebral fracture and symptoms for less than 6 weeks?

- Physical appointment with clinical assessment only
- Physical consultation with X-LWK/X-TWK, depending on location of fracture
- Physical consultation with X-total spine
- Telephone consultation

*Display this question: If are the following elements part of…= referral*
Q26. To which medical specialist do you refer as part of standard treatment for patients with an osteoporotic vertebral fracture and symptoms for less than 6 weeks?

- Geriatric medicine
- Orthopedic surgeon
- Trauma surgeon
- Internist
- Rheumatologist
- Other, namely__________

Q27. In the case of a progression of collapse of an already known osteoporotic vertebral fracture without neurological symptoms, would you deviate from the already established treatment plan?

- Yes
- No
- Other, namely__________

*Display this question: If in case of a progression… = yes*
Q28. On indication, I supplement this standard treatment with: *Multiple answers possible.*

- Pain relief
- Osteoporosis medication
- Physical therapy
- Brace
- Referral to second line
- Surgical treatment
- Other, namely_____________

*Display this question: If on indication, I supplement … = pain relief*Q29. Please specify below which pain relief you supplement standard treatment with on indication. *Multiple answers possible.*

- According to pain ladder WHO
- Only paracetamol
- NSAIDS if there are no contraindications
- Oral opiates
- Transcutaneous opiates
- Other, namely________

*Display this question: If on indication, I supplement … = Osteoporosis medication*
Q30. Please specify below which osteoporosis medication you supplement standard treatment with on indication. *Multiple answers possible.*

- Androgens
- Bisphosphonates
- Bisphosphonates, combination preparations
- Calcium with vitamin D, combination preparations
- Zoledronic acid intravenously
- Calcium regulators: Denosumab, Romosozumab, Teriparitide
- Calcium carbonate
- Estrogen with progesteron, postmenopausal
- Estrogen receptor modulaters
- Estrogens
- Vitamin D and analogs
- Other, namely_________

*Display this question: If on indication, I supplement … = physical therapy*
Q.31 Specify below when you would recommend starting physical therapy

- Start immediately
- Start at a later date

*Display this question: If on indication, I supplement … = brace*
Q32. Specify below which brace you would supplement standard treatment with on indication.

- Lumbar brace
- Dekyphotic brace
- Three-point brace
- Dynamic brace (including Osteolind, Spinova Osteo, Spinomed)
- Other, namely______________

*Display this question: If on indication, I supplement … = referral*
Q33. To which medical specialist and/or outpatient clinic do you refer on indication?

- Referral for fall clinic
- Referral for pain clinic
- Spine specialist referral
- Orthopedic surgeon referral
- Trauma surgeon referral
- Osteoporosis clinic referral
- Internist referral
- Rheumatologist referral
- Other, namely_____________

*Display this question: If on indication, I supplement … = surgical intervention*
Q34. Please specify below which surgical treatment you would use on indication

- Spondylodesis
- Vertebroplasty/kyphoplasty
- Pain treatment (e.g. facet denervation)

Q35. Which of the following options would you see as valuable additions to the current treatment of osteoporotic vertebral fractures?

- None of the following
- More detailed reporting of x-ray diagnostics
- More opportunities for multidisciplinary consultation
- Approachable and open communication first and second line
- Specialized physiotherapy network
- General practitioner assistant specialized in osteoporosis
- More standardized peer reporting
- Other, namely____________

Q36. What is your opinion regarding the alignment around care for patients with osteoporotic vertebral fractures? (Totally disagree, Disagree, Neither disagree nor agree, Agree, Totally agree)

- Within the clinic where I work, the care of patients with osteoporotic vertebral fractures is arranged well
- Care for patients with osteoporotic vertebral fractures is well delineated between first and secondary care
- I feel I have sufficient treatment options available to me
- Responsibilities are sufficiently delineated for the various practitioners within the clinic where I work

Q37. What are the bottlenecks surrounding the care of patients with osteoporotic vertebral fractures?

Q38. Do you have any other comments or additions regarding the care of osteoporotic vertebral fractures?
